# Timely symbiosis: circadian control of legume-rhizobia symbiosis

**DOI:** 10.1042/BST20231307

**Published:** 2024-05-23

**Authors:** Monique Rowson, Matthew Jolly, Suzanna Dickson, Miriam L. Gifford, Isabelle Carré

**Affiliations:** 1 School of Life Sciences, The University of Warwick, Gibbet Hill Road, Coventry CV4 7AL, U.K.; 2 The Zeeman Institute for Systems Biology and Infectious Disease Epidemiology Research, The University of Warwick, Coventry CV4 7AL, U.K.

**Keywords:** circadian clock, legume, *Medicago truncatula*, nitrogen fixation, nodulation, symbiosis

## Abstract

Legumes house nitrogen-fixing endosymbiotic rhizobia in specialised polyploid cells within root nodules. This results in a mutualistic relationship whereby the plant host receives fixed nitrogen from the bacteria in exchange for dicarboxylic acids. This plant-microbe interaction requires the regulation of multiple metabolic and physiological processes in both the host and symbiont in order to achieve highly efficient symbiosis. Recent studies have showed that the success of symbiosis is influenced by the circadian clock of the plant host. Medicago and soybean plants with altered clock mechanisms showed compromised nodulation and reduced plant growth. Furthermore, transcriptomic analyses revealed that multiple genes with key roles in recruitment of rhizobia to plant roots, infection and nodule development were under circadian control, suggesting that appropriate timing of expression of these genes may be important for nodulation. There is also evidence for rhythmic gene expression of key nitrogen fixation genes in the rhizobium symbiont, and temporal coordination between nitrogen fixation in the bacterial symbiont and nitrogen assimilation in the plant host may be important for successful symbiosis. Understanding of how circadian regulation impacts on nodule establishment and function will identify key plant-rhizobial connections and regulators that could be targeted to increase the efficiency of this relationship.

## Introduction

Plants are in constant interaction with an abundance of diverse microorganisms in their environment, with which they can form both antagonistic and mutualistic relationships. These interactions have a significant impact on plant growth, alongside interactions between microbes themselves and environmental factors [1]. One example is the establishment of a mutually beneficial relationship between a photosynthetic host plant and diazotrophic bacteria, resulting in biological nitrogen fixation (BNF). In this process atmospheric nitrogen (N_2_), which is not usable by the plant, is reduced to the biologically-usable ammonia (NH_3_) via the activity of the bacterial enzyme nitrogenase complex. In exchange the plant provides carbon compounds and other beneficial molecules to the bacteria. BNF is a highly significant process as the process to ‘fix’ nitrogen from N_2_ to NH_3_ in industry, the Haber-Bosch process, involves use of extremely high temperature and pressure. In turn this requires enormous fossil fuel use and the process accounts for almost 2% of total world energy requirements according to the UN Food and Agriculture Organization. Therefore, understanding the efficiency of BNF could help to replace industrial fertiliser use to a greater extent in agriculture, as nitrogen fertilisation is a key and limiting factor in crop production.

In many nitrogen-fixing symbioses, specialised root organs called nodules are formed when microsymbionts colonise host plant tissues (e.g. reviewed in [1]). These nodules provide the microaerobic environment required for the activity of the nitrogenase enzyme. In this arrangement, the host plant receives fixed nitrogen in the form of bioavailable ammonia from bacterial symbionts in exchange for photosynthates. There are several examples of mutualistic symbiotic relationships in both legumes and non-legumes [2], but the interaction between leguminous plants and their rhizobial partners is the best studied.

Nitrogen-fixing symbioses are usually established in nitrogen-limited soils. The extent of nitrogen fixation in bacteria is finely tuned via the activity of many bacterial regulatory genes in accordance with internal and external environmental levels of N and C primarily; this regulatory network underpins or characterises symbiotic effectiveness [3]. Much of this regulation occurs via metabolic pathways and these are also responsible for promoting ammonia secretion to the plant [2]. On the plant side of the symbiotic interaction there is also a large number of genetic regulators, with the best characterised of these being the transcription factor Nodule Inception (NIN) and the receptor-like kinase SUperNumary Nodules (SUNN). NIN has been characterised as a master regulator of nodulation, from rhizobial infection to the transition to nitrogen fixation and control over nodule number [4]. SUNN acts to control the autoregulation of nodulation pathway which controls nodule numbers and activity with plant nitrogen need and consists of at least two systemic regulatory circuits [5]. Similarly, there is a great deal of coordination of resource partitioning between plant nodules, rhizobia and the rest of the plant [6].

The symbiosis between legumes and rhizobia has a high degree of specificity, meaning that the efficiency of nitrogen-fixation is highly dependent on the compatibility between the two organisms. Incompatibility can manifest itself as early as unsuccessful partner recognition, which may involve untimely plant immune response activation [7]. It can also occur relatively late, even after nodules have been developed, resulting in nodules that do not fix nitrogen [4]. The host plant has a large amount of control in the process of establishment and maintenance of symbiosis [8], but the molecular mechanisms underlying host control are less well known. Recent research in the model legume *Medicago truncatula* amongst other legume species suggests a role for the circadian clock of the plant host [9–12].

## Circadian clock architecture is conserved between legumes and non-legumes

Circadian clocks enable anticipation of daily changes in environmental conditions linked to the rotation of the earth and represent key adaptations to the 24-h periodicity of our environment. These clocks are found in a wide range of organisms including plants, animals, fungi, cyanobacteria [13], and at least some non-photosynthetic bacteria [14]. Circadian clocks are synchronised (or entrained) to day-night cycles via perception of environmental time cues such as light and temperature and control the periodicity of downstream processes by driving rhythmic changes in gene expression. In plants, more than 30% of the transcriptome is expressed rhythmically, leading to daily changes in a wide range of processes, including carbon metabolism, immunity, hormone signalling, stress acclimatisation and morphogenesis [15]. In addition to its role in temporal coordination over the course of the day, the clock enables perception of seasonal changes in daylength, or photoperiod, and ensures that flowering is initiated at the optimal time of the year [16].

**Figure 1.**
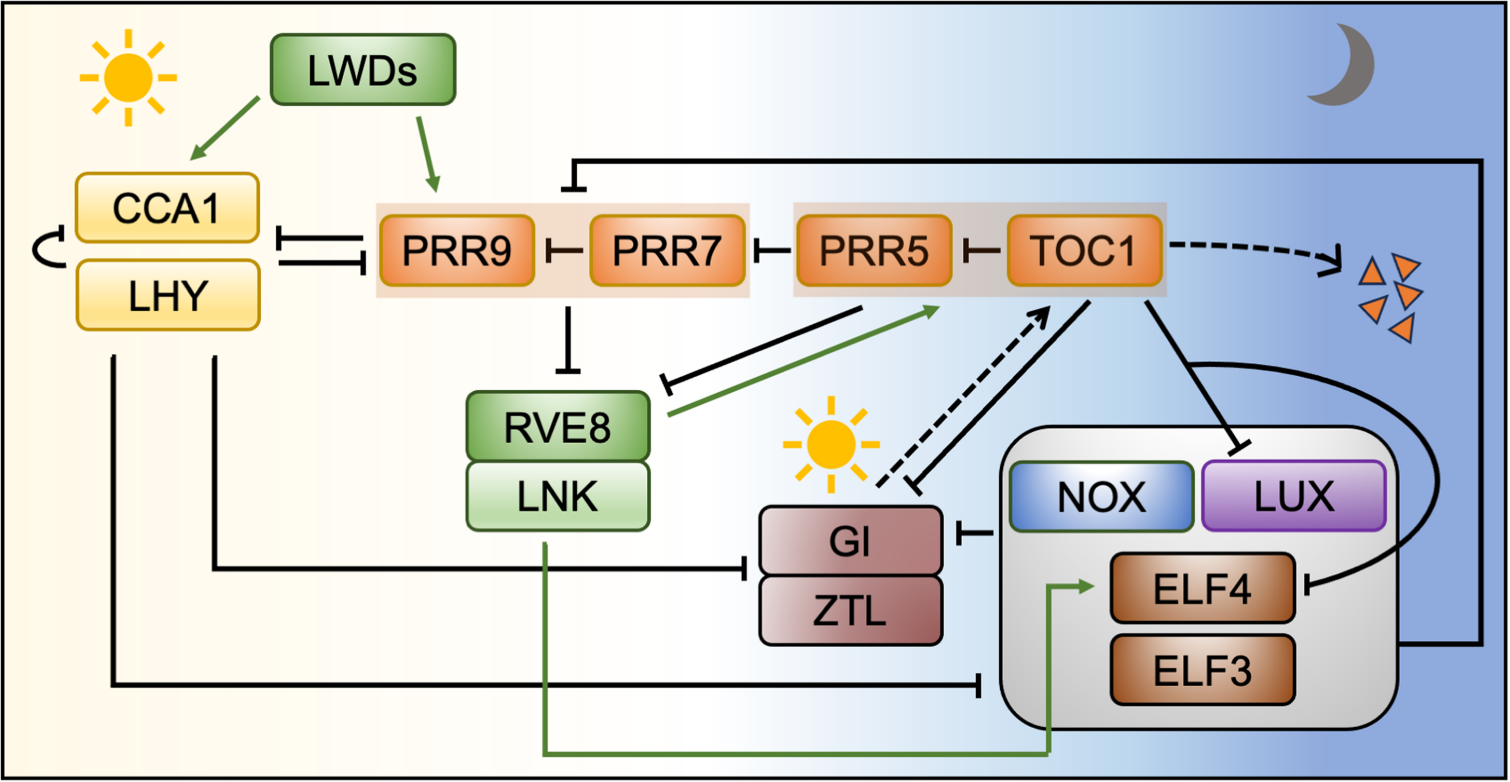
Model of the core circadian oscillator in *A. thaliana*. Yellow background represents the day and blue the night. The core oscillator mechanism is composed of a transcriptional-translational feedback loop. Solid arrows indicate transcriptional regulation, with pointed arrows indicating up-regulation and blunt arrows indicating down-regulation. The MYB transcription factors *CCA1* and *LHY* are expressed in the morning, followed by sequential expression of *PRR* genes in the order *PRR9*, *PRR7*, *PRR5* and *TOC1* (*PRR1*) during the day. These PRR proteins repress *CCA1* and *LHY* expression. An evening complex (EC) consisting of the LUX or NOX, ELF3 and ELF4 proteins forms at night and binds the *PRR9* and *PRR7* promoters at night to inhibit their expression. This relieves the inhibition of *CCA1* and *LHY* transcription and allows the cycle to resume at the following dawn. RVE8, LNK and LWDs are transcriptional activators. Light-dark cycles entrain this central oscillator through multiple mechanisms indicated by the sun-shaped symbols. Light-activation of phytochrome and cryptochrome photoreceptors promotes transcription of *LHY* and *CCA1*, synchronising the rhythms to dawn. The blue light photoreceptor ZEITLUPE (ZTL) partners with GIGANTEA (GI) to control the light-dependent degradation of the TOC1 protein in the evening as indicated by the dashed arrows.

The molecular mechanism of the clock was elucidated in the model non-legume plant *Arabidopsis thaliana* and was shown to be based on a network of transcriptional-translational feedback loops (Figure 1). The central feedback loop is initiated by expression of *CIRCADIAN CLOCK ASSOCIATED 1 (CCA1)* and *LATE ELONGATED HYPOCOTYL* (*LHY)*, in the early morning. These closely related transcription MYB factors repress expression of each other and all other clock components [17]. The reduction in their expression later in the morning enables the expression of *PSEUDO-RESPONSE REGULATOR (PRR)* genes, with *PRR9* and *PRR7* peaking shortly after *LHY* and *CCA1*, *PRR5* around noon and *TIMING OF CAB EXPRESSION 1 (TOC1*, also known as *PRR1*) in the evening. The PRRs repress the transcription of the *CCA1* and *LHY* genes and their sequential expression ensures that this inhibition is maintained until the following morning [18]. This repression is lifted by a third set of genes including *LUX ARRYTHMO (LUX)*, *EARLY FLOWERING 3 (ELF3)* and *ELF4*, which are expressed at dusk and form an evening complex (EC) [19]. The EC represses the expression of the PRR genes, indirectly promoting *LHY* and *CCA1* transcription and allowing the cycle to restart [20]. While the components described so far all function as transcriptional repressors, transcriptional activators also play an important role, with a complex composed of LIGHT-REGULATED WD1 (LWD1) and TEOSINTE BRANCHED1/CYCLOIDEA/PROLIFERATING CELL FACTOR1 (TCP) TCP20 activating expression of *CCA1* in the morning [21], and REVEILLE RVE4 and RVE8 in complex with NIGHT LIGHT–INDUCIBLE AND CLOCK-REGULATED1 (LNK1) and LNK2 acting to promote expression of *TOC1*, *PRR5* and *ELF4* in the evening [22]. The plant clock mechanism is further modulated through post-translational regulation, and the GIGANTEA (GI) protein, expressed in the evening, acts to stabilise the LOV-F box photoreceptor ZEITLUPE (ZTL), resulting in light-dependent degradation of the TOC1 and PRR5 proteins [23]. Circadian clocks are present throughout the plant, but the details of their molecular mechanisms can vary between tissues. For example, in *A. thaliana*, expression of the evening genes *TOC1* and *ELF4* was reported to be rhythmic in shoots but not in shoots [24]. Furthermore, loss of ELF3 function abolished gene expression rhythms in shoots but not in roots [25]. While circadian rhythms in stems and leaves are directly entrained by light-dark cycles through the action of photoreceptors [26], circadian rhythms in roots clocks in root tissues are synchronised to stem tissues via long-distance signalling through transport of sucrose and K^+^ ions [27–29] as well as through light piping from shoots to roots [30]. Shoots also influence circadian rhythms in roots through temperature-dependent transport of the mobile ELF4 protein. ELF4 trafficking from shoots to roots is increased at low temperatures, resulting in a slow-paced root clock, whereas high temperatures decrease the movement, leading to a faster root clock [28].

Most of the clock components identified in *A. thaliana* are conserved across a broad range of plant species, including legumes [31,32]. *M. truncatula* possesses only one homologue of *LHY*/*CCA1*, denoted as *MtLHY*, but contains 7 rather than 5 *PRR* genes (Table 1). Nevertheless, the timing of expression of these clock genes is very similar to that of their *A. thaliana* orthologues, both in roots and shoots [10], suggesting that the mechanism of the clock is largely conserved. One notable difference is that, in the absence of environmental time cues, the free-running period of circadian rhythms is shorter in *M. truncatula* shoots than roots, and that under diurnal light-dark cycles the phase *of M. truncatula* rhythms in roots is advanced relative to shoots [39]. The opposite was found to be the case in *A. thaliana* [27]. Shorter circadian periods in *M. truncatula* roots may be linked to slower ELF4 trafficking from shoots to roots, but this will require further investigation. It will also be important to investigate the functional relevance of having a faster root clock. Shorter period lengths under free-running conditions are generally associated with advanced timing of circadian rhythms under diurnal environmental conditions [40], so one hypothesis is that this is important for optimal timing of metabolic rhythms in roots and/or nodules relative to shoots.

**Table 1.** Clock gene identification in *M. truncatula*

*A. thaliana*		*M. truncatula* potential ortholog		Protein sequence homology (BlastP)			Loss of function phenotype	References
Clock gene name	Locus ID (TAIR10)	Locus ID (Mt4.0v1/MtA17_5.1.9)	Gene name	Reciprocal similarity (%)	Reciprocal similarity score	Reciprocal E value		
*CCA1*	At2g46830	Medtr7g118330/MtrunA17_Chr7g0276421	*MtLHY*	38.7	999	4.9E−126	Fewer and smaller nodules; short period rhythms of leaf movement.	[9,10]
*LHY*	At1g01060		* *	44.7	1296	6.6E−170		
*LUX*	At3g46640	Medtr4g064730/MtrunA17_Chr4g0031961	*MtLUX*	47.5	635	3.4E−81	Fewer nodules; altered leaf movements; Phase advance and dampening rhythms of gene expression in leaves under constant light.	[11]
*NOX*	At5g59570		* *	52.4	561	2.20E−70		
*ELF3*	At2g25930	Medtr3g103970/MtrunA17_Chr3g0135361	*MtELF3a*	32.7	903	1.4E−111	Early flowering (chickpea and pea); Reduced amplitude of clock gene expression (pea).	[33,34]
* *		Medtr1g016920/MtrunA17_Chr1g0151881	*MtELF3b*	35	778	1.2E−93		[35]
		Medtr8g015480/MtrunA17_Chr8g0341441	*MtELF3-like*	29.4	566	1.2E−62		[35]
		Medtr8g015470/MtrunA17_Chr8g0341431	*MtELF3t*	63.4	149	8.2E−13		[36]
*ELF4*	At2g40080	Medtr3g070490/MtrunA17_Chr3g0113431	*MtELF4*	67.9	282	2.0E−33		[35]
*TOC1/PRR1*	At5g61380	Medtr3g037390/MtrunA17_Chr3g0091641	*MtTOC1a*	46.7	1212	3.4E−160	Increased nodule number (soybean).	[12]
* *		Medtr4g108880/MtrunA17_Chr4g0061021	*MtTOC1b*	50.3	1345	4.3E−180		
		Medtr3g037400/MtrunA17_Chr3g0091661	*MtTOC1t*	53.7	247	1.5E−22		[36]
*PRR3*	At5g60100	Medtr4g061360/MtrunA17_Chr4g0030271	*MtPRR3*	43.8	809	5.8E−99		[9]
*PRR5*	At5g24470	Medtr3g092780/MtrunA17_Chr3g0127941	*MtPRR59a*	38.2	883	2.7E−110	Early flowering (in quadruple *M. truncatula* mutant).	[37]
*PRR7*	At5g02810	Medtr1g067110/MtrunA17_Chr1g0181811	*MtPRR7*	47.7	1449	0		
*PRR9*	At2g46790	Medtr7g118260/MtrunA17_Chr7g0276361	*MtPRR59c*	38.3	500	1.7E−56		
* *		Medtr8g024260/MtrunA17_Chr8g0345901	MtPRR59b*	33.5	490	9.6E−55		
*GI*	At1g22770	Medtr1g098160/MtrunA17_Chr1g0201461	*MtGI*	74.9	4445	0	Early flowering under long-days (soybean).	[38]
*RVE8*	At3g09600	Medtr1g067000/MtrunA17_Chr1g0181771	*MtRVE8*	62.2	896	2.7E−121		[36]
*LNK1*	At5g64170	Medtr2g008750/MtrunA17_Chr2g0279081	*MtLNK1/2a*	31.5	534	2.1E−59		This study
* *		Medtr4g094248/ MtrunA17_Chr4g0050491	*MtLNK1/2b*	32.2	530	9.9E−60		This study

Protein sequence similarity calculated with Uniprot Reciprocal BlastP search performed using UniProt v2.12.0. Information about loss of function phenotype is from *M. truncatula* unless otherwise stated.

## Altered function of the circadian clock is associated with disrupted nodulation

Consistent with their proposed roles in the *M. truncatula* circadian clock, mutations at the *MtLHY* and *MtLUX* loci were associated with altered circadian rhythmicity. A transposon insertion in the *MtLHY* gene was shown to result in a 2–4 h advance in expression of the *TOC1*, *LUX*, *PRR5/9* and *CIRCADIAN RHYTHM, AND RNA BINDING 2* (*CCR2*) genes, altered timing of nyctinastic leaf movements and early flowering under 16L8D light (L)-dark (D) cycles [9]. Under constant conditions, *mtlhy* insertion mutants exhibited a shortened period of leaf movements [10]. On the other hand, loss of *MtLUX* function led to rapid dampening of *LHY*, *TOC1*, *PRR5/9*, *PRR7*, *GI* and *CCR2* gene expression rhythms following transfer to constant conditions [11]. *mtlux* mutants also flowered early under short photoperiods (8L16D), suggesting that their photoperiodic response was altered.

Retrotransposon Tnt insertion at the *MtLHY* locus impaired *M. truncatula* ability to form successful symbioses with its symbiont, *Sinorhizobium meliloti*. When inoculated with this rhizobial strain, *mtlhy* mutant plants formed ∼30% fewer nodules [9] and had reduced nodule weight relative to the wild-type [10]. Nodule morphology was altered with fewer meristems in the mutants [10]. This was associated with reduced overall plant weight, both fresh and dry, under low nitrogen conditions [9,10]. In contrast, mock-inoculated plants showed no obvious phenotype, suggesting that the lower biomass of *mtlhy plants* was linked to reduced nitrogen fixation by rhizobia. Similar to *mtlhy* mutants, *mtlux* mutants also had reduced numbers of nodules, suggesting that symbiotic nitrogen fixation was also disrupted by the mutation [11]. In soybean, loss of function of the *GmTOC1b* gene resulted in increased number of nodules, whereas its overexpression inhibited nodulation [12].

The fact that mutations in distinct components of the plant circadian clock impact on nodulation processes suggests that disruption of circadian timing is the primary cause of failure of symbiotic interactions in these mutants, rather than a developmental effect of these mutations. More research is necessary, however, to elucidate the specific stage(s) of nodulation affected by circadian clock disruption and delineate any developmental vs. plant-microbe related effects. Aberrant circadian clock function in the plant host may impair the recruitment of the rhizobial symbiont to the plant root. Alternatively, it may disrupt symbiosis at a later stage, either by causing mis-expression of genes with roles in nodule development, or by altering the temporal coordination of metabolic processes between the bacterium and the plant (e.g. as hypothesised in [10]).

## Bacterial recruitment to plant roots and rhizobial entry is shaped by circadian rhythms

Plants shape microbial communities in the rhizosphere by regulating the availability of nutrients through the release of root exudates. Exudates include amino acids, nucleotides, sugars and secondary metabolites [23] and can vary in composition according to the time of the day [24]. This is thought to drive rhythmic changes in bacterial and fungal communities in the rhizosphere [25]. Thus, altered function of the circadian clock through loss of function of At*CCA1* and At*TOC1* was found to alter both the composition of root exudates and their daily changes [24]. Circadian rhythms in the plant host also influence the long-term composition of the rhizosphere microbiome. For example, the *toc1* and *ztl* mutations in *A. thaliana* were associated with altered rhizosphere community structures [26]. This was shown to have a potential impact on plant health as wild-type plants inoculated with *toc1* and *ztl* microbiomes germinated later and were smaller than plants inoculated with a wild-type microbiome. Similarly, altered function of the clock may impact the recruitment of rhizobia to the plant rhizosphere and may already impact symbiotic relationships before they have been initiated.

**Figure 2.**
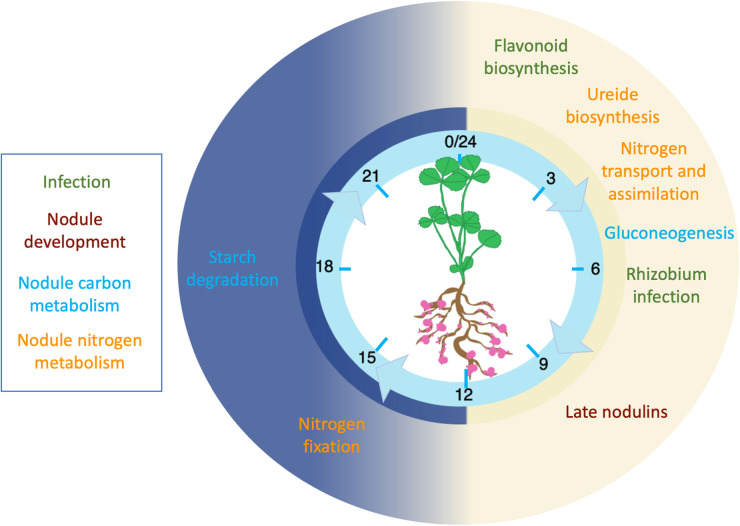
Temporal organisation of nodulation processes in legumes. The diagram illustrates the general understanding derived from multiple plant species. Labels are colour-coded according to processes, as indicated in the box on the top left. Yellow background represents the day and blue the night.

Further clues to the impact of the clock on symbiosis come from studying the molecular signalling dialogue that occurs between host and symbiont. The host plant exudes signalling molecules called flavonoids into the rhizosphere which bind to rhizobial NodD proteins, activating the synthesis of bacterial Nod factors [41]. Nod factors bind to plant Nod factor receptors expressed in root epidermal cells, which leads to initiation of the host nodulation program via the common symbiosis signalling pathway [28]. The plant circadian clock has been found to play a role in modulating several aspects of this symbiosis initiation process (Figure 2). Multiple enzymes with roles in flavonoid biosynthesis were up-regulated in *M. truncatula* nodules in the morning, including the rate-limiting enzyme PHENYLALANINE AMMONIA-LYASE which catalyses the first step in the biosynthesis of polyphenols in plants, including flavonoids, phenylpropanoids and lignin [10]. Furthermore, *FLAVONOL SYNTHASE* (*MtFLS*) expression was significantly down-regulated in *mtlhy* mutants and up-regulated in *MtLHY*-overexpressing plants [31]. These observations suggest that the *M. truncatula* circadian clock can modulate flavonoid synthesis, and that reduced or untimely production of flavonoids may explain the reduced nodulation phenotype of the *mtlhy* mutant. The circadian clock may also modulate other stages of the molecular dialogue between plants and rhizobia, as the *M. truncatula NOD FACTOR PERCEPTION (MtNFP)* gene, which encodes a Nod factor receptor, was expressed rhythmically, peaking in the evening [39]. MtNFP function is essential for Nod factor-inducible responses and its temporal expression pattern may restrict the very first stages of nodulation processes to specific times of the day.

## Aspects of nodule organogenesis are under circadian regulation

Circadian clock disruption may impact the very early stages of nodulation. In soybean, the clock-associated protein *GmTOC1b* was shown to bind the promoters of several key regulators of nodulation to repress their expression [22]. This included including the *NODULE INCEPTION* (*NIN*) transcription factors *GmNIN2a* and *GmNIN2b*, which are required both for infection by rhizobia as well as for nodule formation [42], and the small peptide *EARLY NODULIN 40 (GmENOD40-1)* which in *Lotus japonicus* is required for nodule initiation and subsequent organogenesis [43]. Loss of *GmTOC1b* function resulted in elevated expression of these genes, and this was associated with increased root hair curling and greater numbers of infection threads in response to infection by rhizobia. The *M. truncatula NIN* gene was also expressed rhythmically, peaking in the morning [10].

Bacterial infection occurs in parallel with plant-driven processes such as infection thread formation and nodule development, and this requires coordination between the two partners. The plant immune response must be adjusted to allow colonisation by the symbiont and differentiation into nitrogen-fixing bacteroids [1]. Plant peptides play a key role in this process, supporting rhizobia survival, preventing overgrowth, and simultaneously regulating host compatibility [44]. In *M. truncatula*, a family of over 700 nodule-specific cysteine-rich (NCR) peptides is thought to make a crucial contribution to successful symbiosis by aiding in bacterial adaptation to the intracellular environment [45]. NCR peptides were initially implicated as antimicrobial molecules that limited the reproductive potential of endophytic rhizobia under sub-lethal concentrations [46]. They were later shown to play a role in the metabolic and morphological changes observed in bacteroids in nodules and their diversity in expression levels amongst different legumes plays a large role in the compatibility of symbiotic partners [47]. The expression and sequence of NCR-encoding genes varies between *M. truncatula* accessions and may contribute to differences in symbiotic phenotypes [25].

The expression of NCRs has also been linked to the clock in *M. truncatula* (Figure 2). It was found that 45 NCR genes exhibit rhythmic expression under constant conditions, with 12 of these expressed in the morning, 22 in the evening and 11 in the late night [10] (Figure 2). The MtLHY binding site, also known as the evening element (EE, consensus AGATATTT) was over-represented in the promoters of rhythmic NCRs, and 89 non-rhythmic NCRs also contained EEs in their promoters, suggesting that LHY may directly regulate the timing or level of their expression [10]. Expression of *DEFECTIVE IN NITROGEN FIXATION (MtDNF1)*, a nodule-specific signal peptidase complex responsible for transporting NCR peptides, was also found to be rhythmic in *M. truncatula* with peak expression at dusk [48]. The circadian clock may therefore influence the establishment of symbiotic nitrogen fixation by controlling the expression and transport of these NCR peptides [10]. However, NCR peptides are absent or present in very low numbers from other legumes such as soybean and Lotus spp. and a comprehensive understanding of their function is still lacking [49]. If the rhythmic expression of NCRs is important to the success of symbiotic interactions with rhizobia, this mechanism will not apply in its entirety to all legumes.

## Nodule developmental processes are under circadian regulation

The established nodule of *M. truncatula* comprises infected host cells containing hundreds of membrane-derived symbiotic organelles named symbiosomes, housing thousands of nitrogen-fixing rhizobia [50]. Nodule development requires coordination of a complex network of transcription factors and the action of multiple phytohormones including cytokinins, gibberellins, and auxins [51] and ∼200 genes have been functionally characterised as integral to symbiotic nitrogen fixation [1]. The nodule environment is anaerobic which is required to facilitate the activity of rhizobial nitrogen-fixing enzyme, nitrogenase. This is achieved through establishment of an oxygen-diffusion barrier [52] as well as the expression of oxygen-scavenging proteins known as leghaemoglobins. The latter acts to maintain nodules at micro-oxic levels whilst providing sufficient oxygen to bacteria for respiration and ATP production [53]. Many genes involved in nodule development show rhythmic expression in *M. truncatula* root and shoot tissue [39] (Figure 2). For example, *NODULATION SIGNALLING PATHWAY 2* (*MtNSP2)*, a transcription factor crucial in nodule formation has peak expression midday. *CORYNE* (*MtCRN*) encoding a receptor kinase and *CLAVATA 2* (*MtCLV2I)* encoding a receptor-like protein, together perceive CLAVATA3 signalling peptide which acts to promote cell differentiation, both peak in the late evening [39]. Mis-expression of these genes as a result of impaired clock function would be expected to impact on nodule formation and further work is ongoing characterise this impact.

## Temporal coordination of metabolism between host and symbionts

There is evidence that symbiotic nitrogen fixation exhibits diurnal rhythmicity. For example, in peas, nitrogen fixation was found to be highest at dusk and minimal at dawn. Soluble nitrogen accumulated in nodules during the night and was exported to other parts of the plants through the sap in the morning [54]. In soybean nodules, expression of the rhizobium gene *NifH*, which encodes a component of the nitrogenase enzyme, and that of *FixU*, which plays a role in the biosynthesis of its iron-molybdenum cofactor, peaked in the evening, consistent with nitrogen fixation taking place during the night. Furthermore, expression of a rhizobial glutamine synthetase gene (GlnA1) peaked at dawn, suggesting that the nitrogen fixed at night into ammonia was assimilated into amino acids in the morning [55].

Many genes with roles in nitrogen transport and metabolism are also expressed rhythmically in plant tissues. For example, several nitrate, nitrite, ammonium and amino-acid transporters were found to be expressed in *M. truncatula* nodules around dawn, consistent with plant uptake of nitrogen in the morning [10]. Glutamine synthetase, which catalyses the first step of N assimilation, was also expressed in the morning, as were genes with roles in metabolism of glutamate and other amino acids and in biosynthesis of ureides, a long-distance form of nitrogen transport. The circadian clock of the plant host may therefore play an important role to allow anticipation of nitrogen availability in nodules in the morning and ensure its timely assimilation and transport to other organs.

**Figure 3.**
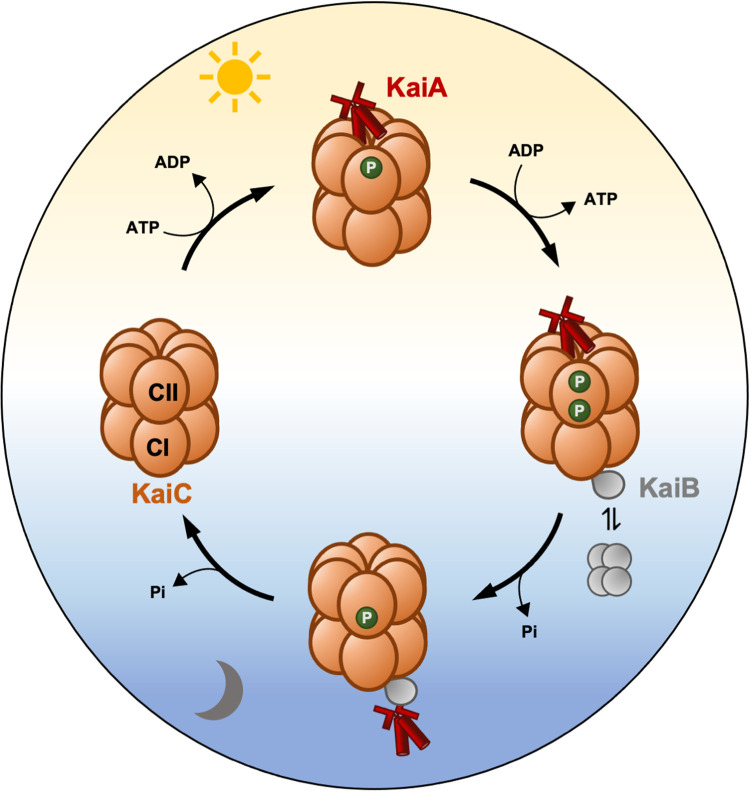
The KaiC protein functions at the core of the circadian clock in cyanobacteria. The circadian oscillator in *Synechococcus elongatus* is composed of three proteins, Kai A, Kai B and KaiC. KaiC is a hexameric protein with each monomer composed of two domans, CI and CII. KaiA binds the CII domain of KaiC during the day and promotes its autophosphorylation. KaiB binds the CI domain of phosphorylated KaiC during the night and this is associated with KaiC dephosphorylation. Dephosphosphorylation of KaiC leads to the release of KaiA, then of kaiB, enabling the cycle to restart at the following dawn. Light sensing proteins are not known to directly interact with and modulate this central mechanism, but metabolic entrainment occurs instead. The ATP:ADP ratio peaks at midday, coinciding with maximum photosynthetic output. This drives the autophosphorylation of KaiC, as indicated by the sun-shaped symbol. The central oscillator also responds oxidation of quinones when photosynthesis ceases at dusk, as indicated by the moon symbol. Yellow background represents the day and blue the night.

What may be the mechanism underlying diurnal changes in nitrogen fixation in the symbiotic bacteria? Rhythmic expression of rhizobial nitrogen fixation genes may be a direct response to the rhythmicity of the plant host, whether to rhythmic changes in availability of certain metabolites or to rhythmic expression of specific signalling molecules — but an alternative hypothesis is that the bacterial symbiont may have a circadian clock of its own. While circadian clocks were long thought to be absent from prokaryotes other than cyanobacteria, recent evidence suggests that some non-photosynthetic bacteria exhibit circadian rhythmicity [14]. For example, the gut bacterium *Klebsiella aerogenes* showed rhythmic expression of a *PmotA::luxCDABE* reporter gene with periods ranging from 22 to 28 h [56]. Transcriptional rhythms were also observed in *Bacillus subtilis*, and these showed the canonical properties of circadian rhythms including entrainment to light-dark cycles, free-running rhythmicity in the absence of environmental time cues, and temperature compensation [57]. The underlying timing mechanism is not known, as neither species contain homologs of known clock genes from other organisms. However, the *Sinorhizobium meliloti* genome encodes a protein with 51% identity with the clock protein KaiC from *Synechococcus elongatus*, which may play a role in either host-driven or free-running rhythmicity. The cyanobacterial circadian clock is made up of three proteins KaiA, KaiB and KaiC. Unlike eukaryotic clocks, it does not utilise transcription-translation feedback loops but instead revolves around the KaiA- and KaiB-driven post translational phosphorylation and dephosphorylation of the KaiC protein (Figure 3). It is not known whether this cycle can take place in the absence of KaiA and KaiB, but hourglass-type rhythms have been observed in the purple non-sulfur alpha-proteobacterium *Rhodopseudomonas palustris* which contains KaiC and KaiB proteins but lacks a KaiA homolog [58]. It is therefore possible that rhizobia also possess proto-circadian timekeeping mechanisms based on KaiC alone [59], and this will be a key direction for future research. It will be important to investigate whether the rhizobial symbiont maintains its own self-sustained rhythms and whether these rhythms are synchronised to environmental signals or to rhythms in the plant host. Temporal coordination of metabolic rhythms between rhizobia and their plant host is likely to be important for successful symbiotic nitrogen fixation. This research may therefore lead to novel approaches for development of optimised rhizobial inoculants.

## Conclusion

Recent studies have shown that domestication of various crop species including tomato, barley required alterations to the clock mechanism [31]. There is emerging evidence, however, that altered clock function can impact on interactions with microorganisms, and that this results in altered rhizosphere communities [60,61]. It is crucially important to understand the consequences for plant health and productivity.

The circadian clock of legume crops was also altered as part of the domestication process. While wild soybean (*Glycine soja*) evolved in northern latitudes, elite cultivars of cultivated soybean (*Glycine max*) were found to exhibit a latitudinal cline in circadian period, suggesting that breeding for optimised performance in these different environments had selected for distinct alleles of clock genes [62]. Soybean varieties that are adapted to tropical regions have a long juvenile, late flowering phenotype caused by a mutation in the *ELF3* gene [63]. Nothing is known about the consequences for symbiotic nitrogen fixation, however. Further research is necessary to better understand how daily rhythms in both the plant host and in the rhizobium symbiont influence nodule formation. This could enable us to breed legumes or to engineer rhizobial strains that can form efficient nitrogen-fixing nodules over a range of latitudes. This has the potential to greatly lower the requirement for nitrogen fertilisers in agriculture and to reduce their negative impact on the environment, bringing broad benefits.

## Perspectives

- Links between the circadian clock and plant growth and development have long been known, but more recently the plant circadian clock was shown to affect interactions between plants and microbes which have key benefits for plant productivity and for the soil and environment.
- *Summary of the current thinking*: study of legumes and of their highly orchestrated interactions with soil-borne, nitrogen-fixing rhizobia have uncovered circadian rhythmicity in all aspects of these symbioses, from early microbial recognition to nodule development and later metabolic exchanges. Altered circadian rhythmicity in the plant host was found to lead to disrupted nodulation, suggesting that appropriate circadian regulation was key to the success of symbiotic nitrogen fixation.
- *Comment on future directions*: future work will investigate how the rhythmic expression of plant nodulation genes impacts on nodule formation. There is also evidence that rhizobium symbionts exhibit rhythmic gene expression and investigation of the underlying mechanism may identify useful targets for optimisation of symbiotic nitrogen fixation.

## Abbreviations

BNF: biological nitrogen fixation
EC: evening complex
EE: evening element
*MtNFP*: *M. truncatula NOD FACTOR PERCEPTION*
NCR: nodule-specific cysteine-rich
SUNN: SUperNumary Nodules
